# Production of TiC-MMCs Reinforcements in Cast Ferrous Alloys Using In Situ Methods

**DOI:** 10.3390/ma14175072

**Published:** 2021-09-04

**Authors:** Aida B. Moreira, Laura M. M. Ribeiro, Manuel F. Vieira

**Affiliations:** 1 Department of Metallurgical and Materials Engineering, University of Porto, R. Dr. Roberto Frias, 4200-465 Porto, Portugal; up201108098@fe.up.pt (A.B.M.); lribeiro@fe.up.pt (L.M.M.R.); 2 LAETA/INEGI—Institute of Science and Innovation in Mechanical and Industrial Engineering, R. Dr. Roberto Frias, 4200-465 Porto, Portugal

**Keywords:** casting process, in situ synthesis, locally reinforcement, metal matrix composite, titanium carbide

## Abstract

This literature review aims to summarize the research conducted on the production of locally reinforced ferrous castings based on metal matrix composites reinforced with TiC (TiC-MMCs). One way to improve the wear resistance of cast components is to reinforce critical regions locally with metal matrix composites (MMCs) without changing the toughness of the component core. The in situ method of self-propagating high-temperature synthesis is one of the main approaches for the production of this enhanced material. Using this approach, the reinforcement is formed from a powder compact inserted in the mold cavity. The temperature of the liquid metal then produces the combustion reactions of the powders, which promote the formation of the ceramic phase. This paper focuses on eight powder systems used to synthesize TiC: Ti-C, Ni-Ti-C, Ni-Ti-B_4_C, Fe-Ti-C/Fe-Cr-Ti-C, Cu-Ti-B_4_C, Al-Ti-C, and Al-Ti-B_4_C, and provides an overview of the methodologies used as well as the effect of processing variables on the microstructural and mechanical characteristics of the reinforcement zones.

## 1. Introduction

Abrasive wear is still one of the main causes of the failure of equipment, machines, and industrial components. It, therefore, remains the focus of scientific and applied research on high wear performance materials. The cost of abrasive wear has been estimated as being up to 1–4% of the gross national product for industrialized countries, and its impact is particularly evident in industrial activities, including mining, land, rock, and minerals processing and handling [1,2,3,4,5,6,7].

Abrasive wear mechanisms can involve both plastic deformation and brittle fracture of the material. The plastic deformation is mainly determined by the hardness, while the brittle fracture also depends on the toughness of the material. This means that other properties beyond hardness, such as elastic modulus, yield strength, fracture toughness, as well as microstructure, and chemical composition play a prominent role in wear abrasion resistance [2,3,4]. Thus, a lot of different materials have been used in wear performance demand applications. High-speed steels (1–1.6% C), cold-work and hot-work tool steels (≈1% C), martensitic steels (≈0.7% C), pearlitic Cr-Mo steels (≈1% C), austenitic manganese steels (12–14% Mn and ≈1% C) and Cr and high-Cr white cast irons (WCIs) are among the metallic materials most often used in abrasive wear conditions: They represent a good compromise between the wear resistance and toughness [1,2,3,4].

The production of high hardness steel and cast iron components by heat-treatment and alloying may not be the best way to ensure high wear resistance because of the associated low toughness and resistance to dynamic loading. Other factors, such as cost and overall weight, may also restrict their application. An alternative approach is to locally reinforce specific regions of the component that will be exposed to wear in service while maintaining the properties of the component core, in particular the toughness and ductility [3,6,8,9,10,11,12]. The production of the reinforcement uses a simple methodology based on the formation of local metal matrix composites (MMCs) with ceramic particles. Two different approaches can be used: the so-called ex situ, where the ceramic particles are previously produced with the desired shape and introduced in the mold cavity before metal casting [5,12,13,14,15,16,17,18], and the in situ approach that uses a mixture of metallic and non-metallic powders previously compacted and then inserted in the mold, where they react together through combustion reactions due to the effect of the liquid metal temperature and, consequently, produce the composite reinforcement [9,19,20,21,22,23,24].

Self-propagating high-temperature synthesis (SHS) is the main in situ technique used to reinforce ferrous cast components. This technique is based on a combustion reaction that is initiated by the heat of the liquid metal, resulting in the synthesis of a ceramic compound [6,23,25,26,27]. The heat released from the combustion should be sufficient to sustain the reaction until the reagents have been consumed.

According to several studies [28,29,30], SHS process involves the following steps:

1. Mixture and compaction of powders at room temperature or slightly higher;
2. Start of combustion (ignition or auto-ignition);
3. Self-propagating combustion, which leads to the formation of ceramics (carbides, borides, and silicides) or intermetallic compounds (e.g., TiAl).

The chemical reactions are thermally activated. For this, two methods are used: heating the entire compact or igniting it locally. In the first method, usually called volume combustion synthesis or thermal combustion synthesis, the temperature is uniform over any cross-section of the compact and so the reaction occurs uniformly throughout the powders. In the second case, the ignition occurs in a small volume of material (≈1 mm^3^) and then propagates itself in the form of a combustion wave. Thus, this method is called wave combustion or auto-wave combustion [23,29,31].

The combustion synthesis can be described by the reaction:

$$A+\mathrm{xB}\to {\mathrm{AB}}_{x}$$

where A is usually a metal (Co, Cu, Fe, Hf, Mo, Nb, Ni, Si, Ta, Ti, Zr), B a non-metal (B, C, Si) or a metal (Al, Ni), and AB_x_ a carbide, boride, silicide, or intermetallic compound [29,32,33]. This reaction is characterized by an adiabatic temperature (T_ad_), which is an indicator of the combustion front temperature [28,29,32]. As shown in Table 1, the T_ad_ can be quite high, as in the case of TiC and TiB_2_.

In general, the minimum T_ad_ for self-sustaining combustion is 2200 °C [35,36], although, for temperatures between 1200 °C and 2200 °C, self-sustaining combustion can be activated by preheating the reagents or applying mechanical energy to the system. It has been empirically determined that 1530 °C is enough to initiate these reactions [32].

The heat of formation is also used to assess the self-sustainability of the reaction front. All the compounds with the heat of formation higher than 150 kJ∙mol^−1^ can be synthesized by this method. When the heat of formation is close to 100 kJ∙mol^−1^, it is necessary to preheat the reagents in order to self-sustain the reaction front. The synthesis by self-sustaining combustion has not been observed for compounds with the heat of formation lower than 100 kJ∙mol^−1^ [29,33].

The main advantages of SHS, compared to conventional sintering of ceramic reinforcements, include lower energy requirement, shorter reaction time, fabrication of reinforced products in a single process, and simpler and more affordable equipment. However, the SHS process can be difficult to control due to the high reaction rates. This issue can be largely resolved by the addition of specific compounds (usually designated moderators), which do not participate in the reaction but increase the thermal mass of the system and, consequently, decrease the T_ad._ Another strategy is to increase the particle size of the powders, resulting in a decrease in the combustion front temperature [32,35].

Several powders systems can be used to reinforce ferrous components by the casting–SHS process. Depending on the powder system, the SHS process proceeds by a single combustion reaction or by more complex reaction paths involving the formation of intermediate phases.

According to Rogachev and Mukasyan [29], the combustion synthesis of TiC in the Ti-C system is as follows:

$$\mathrm{Ti}+C\to \mathrm{TiC}+310\;\mathrm{kJ}\cdot {\mathrm{mol}}^{-1}$$

Relating to Ni-Ti-C system, Zhu et al. [21] described the synthesis of TiC with the following combustion reaction:

Ti+C+xNi→TiC+xNi+Q

For the same system, other exothermic reactions were identified by Zhu et al. [21] and Jie-Cai et al. [37]:

$$2\mathrm{Ti}+\mathrm{Ni}\to {\mathrm{Ti}}_{2}\mathrm{Ni}+83\;\mathrm{kJ}\cdot {\mathrm{mol}}^{-1}$$

$$\mathrm{Ti}+\mathrm{Ni}\to \mathrm{TiNi}+67\;\mathrm{kJ}\cdot {\mathrm{mol}}^{-1}$$

$$\mathrm{Ti}+3\mathrm{Ni}\to {\mathrm{TiNi}}_{3}+140\;\mathrm{kJ}\cdot {\mathrm{mol}}^{-1}$$

Concerning the Ni-Ti-B_4_C system, Yang et al. [38] described the overall reaction of synthesis of TiC and TiB_2_ and Yang et al. [39] detailed the combustion reaction path, respectively as follows:

$$\mathrm{xNi}+3\mathrm{Ti}+B_{4}C\to 2{\mathrm{TiB}}_{2}+\mathrm{TiC}+\mathrm{xNi}$$

$$\mathrm{Ni}+\mathrm{Ti}+B_{4}C\to {\mathrm{Ti}}_{2}\mathrm{Ni}+{\mathrm{Ni}}_{2}B+{\mathrm{Ni}}_{4}B_{3}+\mathrm{TiC}+\mathrm{Ni}+\mathrm{Ti}+B_{4}C\to \mathrm{Ni}-\mathrm{Ti}\left(l\right)+\;\mathrm{Ni}-B\left(l\right)\to \mathrm{Ni}-\mathrm{Ti}-C\left(l\right)+\mathrm{TiC}\to \mathrm{Ni}-\mathrm{Ti}-C-B\left(l\right)\to \mathrm{TiC}+{\mathrm{TiB}}_{2}+\mathrm{Ni}$$

Previous studies [24,40] described the overall reaction of synthesis of TiC in the Fe-Ti-C system as follows:

$$\mathrm{Ti}+C+\mathrm{xFe}\to \mathrm{TiC}+\mathrm{xFe}+184\;\mathrm{kJ}\cdot {\mathrm{mol}}^{-1}$$

In those studies, two main routes are suggested:

$$a)\;\mathrm{Ti}+C\to \mathrm{TiC}\;∆G^{0}=-186,600+13.22T\;\left(J\cdot {\mathrm{mol}}^{-1}\right)$$

$$b)\;2\mathrm{Fe}+\mathrm{Ti}\to {\mathrm{Fe}}_{2}\mathrm{Ti}\;∆G^{0}=-87,450+10.73T\;\left(J\cdot {\mathrm{mol}}^{-1}\right),$$

$${\mathrm{Fe}}_{2}\mathrm{Ti}+C\to 2\mathrm{Fe}+\mathrm{TiC}\;∆G^{0}=-99,150+2.49T\;\left(J\cdot {\mathrm{mol}}^{-1}\right)$$

Two studies [41,42] on the Cu-Ti-B_4_C system, described the overall reaction of synthesis of TiC and TiB_2_ as follows:

$$\mathrm{xCu}+3\mathrm{Ti}+B_{4}C\to 2{\mathrm{TiB}}_{2}+\mathrm{TiC}+\mathrm{xCu}$$

For the same system, other reactions were also identified:

$$\frac{3}{4}\mathrm{Ti}+\frac{1}{4}B_{4}C\to \frac{1}{2}{\mathrm{TiB}}_{2}+\frac{1}{4}\mathrm{TiC}$$

$$\frac{5}{6}\mathrm{Ti}+\frac{1}{6}B_{4}C\to \frac{2}{3}\mathrm{TiB}+\frac{1}{6}\mathrm{TiC}$$

$$\frac{2}{3}\mathrm{Ti}+\frac{1}{3}\mathrm{Cu}\to \frac{1}{3}{\mathrm{Ti}}_{2}\mathrm{Cu}\;∆G^{0}=-12,131+4.688T\;J\cdot {\mathrm{mol}}^{-1}$$

$$\frac{1}{2}\mathrm{Ti}+\frac{1}{2}\mathrm{Cu}\to \frac{1}{2}\mathrm{TiCu}\;∆G^{0}=-11,206+3.272T\;J\cdot {\mathrm{mol}}^{-1}$$

According to Choi and Rhee [43], the overall combustion reaction in the Al-Ti-C system is as follows:

2Ti+2C+xAl→2TiC+xAl

The same authors suggested that the combustion reaction takes place in several steps with the precipitation of titanium aluminides (TiAl_x_):

$$2\mathrm{Ti}+2C+\mathrm{xAl}\to {\mathrm{TiAl}}_{x}+\mathrm{Ti}+2C$$

$${\mathrm{TiAl}}_{x}+\mathrm{Ti}+2C\to {\mathrm{TiAl}}_{x}+\mathrm{TiC}+C$$

$${\mathrm{TiAl}}_{x}+\mathrm{TiC}+C\to \mathrm{TiC}+\mathrm{Ti}+\mathrm{xAl}+C$$

TiC+Ti+xAl+C→2TiC+xAl

Concerning the Al-Ti-B_4_C system, the results of three studies [44,45,46] suggested that TiC and TiB_2_ are synthesized through the following combustion reaction and its partial reactions, respectively:

$$\mathrm{xAl}+3\mathrm{Ti}+B_{4}C\to 2{\mathrm{TiB}}_{2}+\mathrm{TiC}+\mathrm{xAl}$$

$$\frac{3}{4}\mathrm{Al}\left(s\right)+\frac{1}{4}\mathrm{Ti}\left(s\right)\to \frac{1}{4}{\mathrm{Al}}_{3}\mathrm{Ti}\left(s\right)$$

$$\frac{10}{13}\mathrm{Al}\left(s\right)+\frac{3}{13}B_{4}C\left(s\right)\to \frac{6}{13}{\mathrm{AlB}}_{2}\left(s\right)+\frac{1}{13}{\mathrm{Al}}_{4}C_{3}\left(s\right)$$

$$\frac{3}{4}\mathrm{Ti}\left(s\right)+\frac{1}{4}B_{4}C\left(s\right)\to \frac{1}{2}{\mathrm{TiB}}_{2}\left(s\right)+\frac{1}{4}\mathrm{TiC}\left(s\right)$$

$$\frac{3}{4}{\mathrm{Al}}_{3}\mathrm{Ti}\left(s\right)+\frac{1}{4}B_{4}C\left(s\right)\to \frac{1}{2}{\mathrm{TiB}}_{2}\left(s\right)+\frac{1}{4}\mathrm{TiC}\left(s\right)+\frac{9}{4}\mathrm{Al}\left(s\right)$$

$$\frac{1}{2}{\mathrm{AlB}}_{2}\left(s\right)+\frac{1}{2}\mathrm{Ti}\left(s\right)\to \frac{1}{2}{\mathrm{TiB}}_{2}\left(s\right)+\frac{1}{2}\mathrm{Al}\left(s\right)$$

$$\frac{1}{4}{\mathrm{Al}}_{4}C_{3}\left(s\right)+\frac{3}{4}\mathrm{Ti}\left(s\right)\to \frac{3}{4}\mathrm{TiC}\left(s\right)+\mathrm{Al}\left(s\right)$$

The following sections provide an overview of the literature on ferrous cast components locally reinforced with TiC-MMCs or TiB_2_-MMCs produced by in situ methods. Titanium carbide and titanium diboride are considered to be the best ceramic particles for ferrous matrix composites due to their high hardness, excellent wear resistance, good wettability, and stability in ferrous matrices [44,47]. A summary of the studies reported in the literature is organized according to the types of powder systems used: Ti-C, Ni-Ti-C, Ni-Ti-B_4_C, Fe-Ti-C/Fe-Cr-Ti-C, Cu-Ti-B_4_C, Al-Ti-C, and Al-Ti-B_4_C. Special attention is given to the effect of processing parameters, such as size and content of each type of powder, binders used, compaction pressure, and casting temperature, on the microstructure and mechanical properties.

## 2. Cast Ferrous Alloys Reinforced by SHS

**Figure 1:**
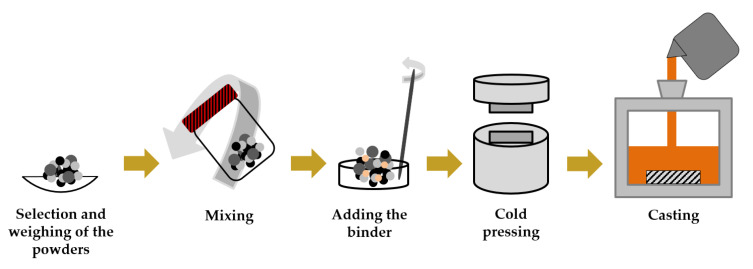
Scheme of the fabrication steps for the production of a reinforced part (adapted from [64]).

**Figure 2:**
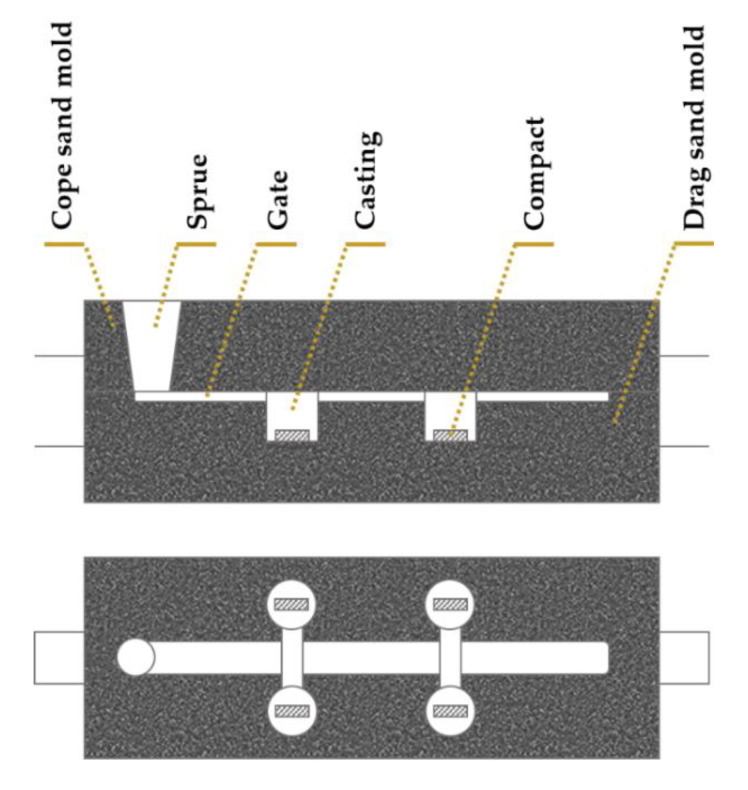
Schematic view of the positioning of the compacts in drag mold (front and top views).

The application of the SHS method to casting enables the fabrication of locally reinforced ferrous components through an efficient, low cost, and simple process. The main steps in this fabrication method are shown in Figure 1. First, the powders are selected, weighed, mixed and homogenized. After the compaction of the powder mixture by cold-pressing using a metallic mold, the compacts are placed and fixed in the drag mold, as shown in Figure 2. Then, the molten metal is poured, obtaining the reinforced samples at the end of the cooling.

It is possible to apply the SHS technique to investment casting, and vacuum expendable pattern casting (V-EPC) technology [48,49]. In these two cases, the outer surface of the composite must have a low roughness in order not to compromise the quality of the surface of the components produced.

**Figure 3:**
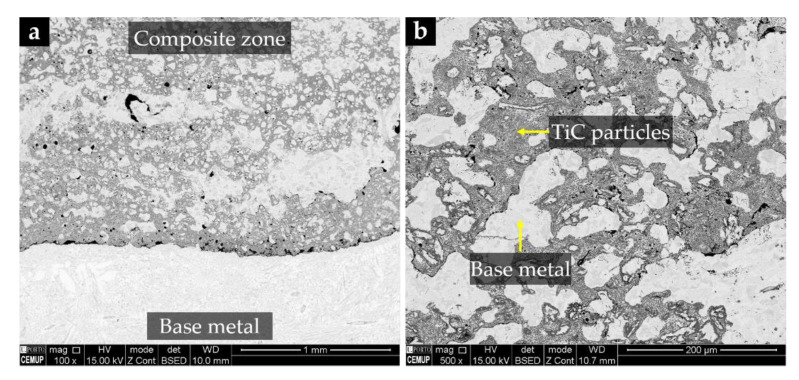
SEM-BSE images of the microstructure of a reinforcement produced in a high-Cr white cast iron using the Al-Ti-C powder system: (**a**) bonding zone and (**b**) metal matrix composite zone with TiC particles.

Many types of powders can be processed by SHS but much of the research on MMCs fabricated using this technique has been focused on the following systems: Ti-C [50,51,52,53,54,55,56,57], Ni-Ti-C [58,59], Ni-Ti-B_4_C [38,60,61], Fe-Ti-C/Fe-Cr-Ti-C [7,10,20,22,24,48,54,62], Cu-Ti-B_4_C [6,26,41,42,63], Al-Ti-C [43,64] and Al-Ti-B_4_C [44,65]. The studies carried out have contributed to the understanding of the effect of key factors, such as particle size, compaction pressure, and casting temperature on the final microstructure and mechanical properties of the MMCs. Figure 3 illustrates the microstructure of a TiC-MMC produced using the Al-Ti-C powder system.

As evident from the literature review, the first cases of SHS application on cast ferrous alloys are from 2005, and the majority of the applications were developed in steel parts [6,20,22,44,48,58], using a pressureless infiltration technique, which is less frequent in cast iron components [24,53,54,64]. The main findings on using the most common powder systems will be presented in the following five sections.

## 3. Ti-C System

Fraś et al. [51] developed a surface reinforcement with TiC in cast steel specimens. Particles with a size range of 0.5 to 4.0 µm and agglomerated particles with 20 µm were observed. The same authors found, in another study [55], that the TiC agglomerates and the oval shape of TiC particles result from high local temperature due to the SHS reaction. The hardness of the reinforcement (with a thickness of 550 to 1200 µm) reached 1134 HV and the matrix was close to 588 HV. Olejnik et al. [50] fabricated a reinforcement composed of TiC particles, also in cast steel, with an oval shape and hardness of 696 HV (four times higher than the ferritic steel).

The influence of the compaction pressure on the wear resistance of TiC reinforcements in low carbon steels was also studied [56], and it was concluded that the increase in the compaction pressure from 250 MPa to 600 MPa causes an increase in hardness from 489 HV to 1523 HV and a decrease in the loss of mass during the abrasion test.

More recently, the effect of the binder addition (an aqueous solution of carboxymethylcellulose-CMC) in powder mixtures of Ti (44 µm) and graphite (3 µm) has been investigated by Szymański et al. [57]. The authors reported that the mixture prepared with 10 wt.% aqueous CMC solution showed reduced wettability, and the mixtures with 5 and 2 wt.% generated in situ composites with thicknesses of 1.2 and 1.0 mm, respectively. In these two cases, an inhomogeneous microstructure due to reactive infiltration was found. The hardness of the composites was ≈600 HV 1 and ≈750 HV 1, representing an increase of about 50% and 88% relative to the base metal (≈400 HV 1).

Table 2 provides the relevant information on base materials, experimental conditions, and main results from the literature review on the Ti-C system. The majority of the studies are focused on the reinforcement of steel parts [50,51,52,56,57] and on the effect of the compaction pressure [52,56], nature of the binder [51,57], and type of atmosphere used [51,53], on the microstructure and properties of the reinforcement.

## 4. Ni-Ti-C and Ni-Ti-B_4_C Systems

The few studies on the Ni-Ti-C and Ni-Ti-B_4_C systems [58,59,60,61] permit us to conclude that the granulometric distribution of Ni, Ti, and C particles affects the quality of the interface between the reinforcement and the matrix, particularly the interface porosity, which in turn influences the wear resistance of the reinforcement zone. Yang et al. [58] concluded that small particles of C (≈1 µm) provided a good bonding between the reinforcement and the base metal due to the increase in the contact surface between Ti and C, which increases the reaction rate. The hardness and wear resistance of the reinforcement (≈45 HRC and 0.7152 × 10^−10^ m^3^∙m^−1^) were significantly higher than those of the matrix (≈20 HRC and 2.268 × 10^−10^ m^3^∙m^−1^).

The nickel is used as a binder since, in the liquid state, it decreases the contact angle (30°), improving the wettability of the TiC phase [61,66]. However, Ni may decrease the T_ad_ and the combustion temperature (T_c_), so having a reducing effect on the self-sustaining combustion [32,38]. In this respect, Yang et al. [38] found that the maximum addition of Ni should be 66 wt.% to guarantee the occurrence of the SHS process. In other studies (see Table 3), the percentage of Ni varied from 10 to 40 wt.%.

Wang et al. [61] fabricated reinforcements from the Ni-Ti-B_4_C system. The composite produced is composed of polygonal TiB_2_ particles and spherical TiC particles uniformly distributed. This microstructure was also confirmed by other authors [67,68]. The size of TiC and TiB_2_ is affected by the content of Ni and the authors reported a decrease from 7 µm to less than 1 µm on increasing the Ni content from 10 to 40%. It is suggested that a high Ni content affects the diffusion coefficient of B and C, inhibiting the growth of TiB_2_ and TiC particles, and so a significant hardness increase in the reinforcement could be achieved. The hardest reinforcements were found with 30% and 40% Ni (48 HRC and 47 HRC, respectively). On the other hand, Wang et al. [47], found coarse TiB_2_ particles when the base metal solidified more slowly.

The influence of the reagent powders’ size in the microstructure and mechanical properties of the reinforcement has also been investigated by several authors [58,69]. Yang et al. [69] found that the increase in the size of B_4_C particles delays the beginning of the reaction and decreases the velocity of the combustion wave. The authors also found that the size decrease in Ni particles (from ≈45 µm to ≈3 µm) and Ti particles (from ≈150 µm to ≈25 µm) favors the formation of TiC and TiB_2_ phases.

Table 3 summarizes the main results of the studies done on the application of the Ni-Ti-C and Ni-Ti-B_4_C systems. The main issues investigated are the effect of the initial powders ratio [59,60,61], and the size of the powders [58], on the mechanical properties of the produced reinforcements.

## 5. Fe-Ti-C and Fe-Cr-Ti-C Systems

Fe-Ti-C and Fe-Cr-Ti-C are the powder systems that have been most investigated to date. Since 2006, several studies have been conducted to quantify the effect of Fe, Ti, and C content on the microstructure and mechanical properties of the reinforcements produced.

Concerning the Fe concentration in the powder mixture, the maximum addition was 70 wt.% [20]. However, Zhang et al. [40] indicated a limit of 57 wt.% Fe due to its effect in T_c_ decreasing. Olejnik et al. [54], in turn, concluded that the addition of 50 wt.% Fe reduced the size of TiC precipitates and improved their distribution in the matrix, avoiding the formation of agglomerates; however, the hardness of the composite was reduced. The hardness of the composite made from the mixture without Fe was 742 ± 163 HV (four times the hardness of the base metal), while with 10 wt.% and 50 wt.% of Fe, was 562 ± 29 HV and 256 ± 38 HV, respectively.

Bai et al. [48] concluded that an increase in Fe content reduces the heat released by the SHS reaction, leading to inclusions (resulting from non-reacted powder) and, consequently, a decrease in the wear resistance of the composite. The same authors produced a reinforcement from a powder mixture with 80 wt.% of Ti + C (Ti:C of 4:1) and 20 wt.% of Fe with a hardness of 63 HRC and a wear resistance five times higher than that of the base metal (Mn alloyed steel). In another study [20], powder mixtures without Fe addition resulted in macroporosity, dimensional changes, and partial cracking of the reinforcement zone. Several authors [48,70] suggested that these effects were caused by the increase in the temperature during the SHS reaction, which favors reactive infiltration and gas emission.

The Fe addition to the powder mixture was replaced by ferrous alloys in several other studies. For example, Olejnik et al. [10] investigated the influence of the addition of 70 wt.% of Ni-Hard alloy on the microstructure of the reinforcement produced. A random distribution of sub-micrometer particles of TiC with uniform size and shape was observed. The hardness of the reinforcement (1500 HV 1) was three times higher than that of the base metal (medium-C steel), and the wear resistance was four and five times higher than Mn steel and high-Cr white cast iron, respectively. In a recent study [62], a reinforcement produced from a mixture of Ti, graphite, and 70 wt.% of Hadfield steel powder showed an average hardness of 785 HV, three times higher than the base metal (Mn steel), and a wear rate 98% higher. The authors concluded that the addition of the Hadfield steel contributed to the increase in undercooling during the solidification of the base metal, and, consequently, the decrease in the TiC particles size (≈0.55 µm). The stabilization of austenite in the microstructure of the composite occurred due to the high content of Mn. Olejnik et al. [7] have also studied the effect of the addition of 30 wt.%, 50 wt.%, 70 wt.%, and 90 wt.% of high-Cr white iron powder and found an increase in the dimensional stability and homogeneity of the microstructure with the content increase in the high-Cr white iron powder. The authors verified the association between the lowest wear rate and the maximum addition (90 wt.%) of the white high-Cr iron powder, due to the presence of Cr_7_C_3_ carbides in the austenitic matrix.

The influence of the process parameters, such as powder compaction pressure and casting temperature, on the soundness of the bond interface and properties of the reinforcement, has also been studied [24,48]. Bai et al. [48] noted that a low density of the green compacts improves the infiltration of the base metal, reducing the hardness of the final composite. The best results for hardness (≈63 HRC) and wear resistance (five times higher than that of the base metal) were achieved with a compaction pressure of 200 MPa. He et al. [24], investigated the effect of casting temperature (1450 to 1600 °C) on the microstructure. The authors found that the lower casting temperatures (1450–1500 °C) promote the formation of spherical Fe_2_Ti and TiC particles with a size between 1 and 3 µm, uniformly distributed in the matrix. This microstructure led to the maximum hardness (68 HRC), nearly twice the hardness of the matrix (32–36 HRC). For the highest temperature tested (1600 °C), large agglomerates of TiC have formed, resulting in a lower hardness.

Table 4 and Table 5 present the relevant information on base materials, experimental conditions, and main results of the studies carried out on the application of the Fe-Ti-C and Fe-Cr-Ti-C systems.

## 6. Cu-Ti-B_4_C/Cu-Ti-C Systems

The Cu content affects the self-sustaining combustion reactions that take place on the Cu-Ti-B_4_C/Cu-Ti-C systems. The maximum addition of Cu powder reported in the literature is 72 wt.% and the shortest ignition time is obtained with 20 wt.% Cu [41,42]. Liang et al. [26] studied the Cu-Ti-B_4_C system, mainly the effect of Cu powder content on the microstructure and mechanical properties of the reinforcement produced. They found that the addition of 50 wt.% and 60 wt.% Cu resulted in insufficient infiltration of the compact by the base metal due to a decrease in the maximum reaction temperature. This temperature reduction led to a decrease in the amount and size of TiC and TiB_2_ particles and, consequently, an increase in the wear rate [6,26]. It was also concluded that the hardness (50 HRC) of the reinforcement produced with 10 wt.% Cu was significantly higher than that of the base metal (20 HRC), whereas the addition of Cu 30 wt.% resulted in the lowest wear volume loss. In a recent study [63], the same authors found an increase in the ignition temperature with the increase in the B_4_C particle size (from ≈3.5 µm to ≈150 µm). The combustion temperature decreased, and the size of the TiC and TiB_2_ particles formed in the composite became smaller. The quality of the interface between the reinforcement and the base metal was affected and the porosity increased significantly. Intermediate phases, such as Fe_2_B, were observed in the microstructure of the composite zone, indicating that the combustion reaction was not complete. The reinforcement produced with the smallest B_4_C particles (≈3.5 µm) presented the best wear resistance with a wear mass loss 76% lower than that of the base metal (Mn-steel).

Concerning the effect of C particle size, Liang et al. [73] verified that the weakness of the interface bonding and the porosity of the composite zone increase with the C particles size (from ≈1 µm to ≈150 µm). The formation of the intermediate phase Fe_2_Ti is also associated with the larger C particles due to an incomplete combustion reaction. Finally, the best wear performance was obtained with the smaller particles of C (≈1 µm) showing a volume wear loss 67% lower than the Mn-steel.

Table 6 comprises the results of the studies performed on the application of the systems Cu-Ti-B_4_C and Cu-Ti-C in the reinforcement of steel components. The subjects investigated concern the effect of the percentage of Cu addition [6,26,74], the particle size of C [73], and B_4_C [63] on the hardness and wear resistance of the reinforcements produced.

## 7. Al-Ti-C and Al-Ti-B_4_C Systems

Choi and Rhee [43] verified that the addition of Al (0–40 wt.%) in a mixture with a Ti:C ratio of 1, resulted in the reduction in TiC particle size (from ≈15 µm to 0.4 µm) and porosity of the ceramic composite produced. The authors concluded that Al not only acted as a binder in the Ti-C system, forming a thin layer at the surface of the TiC particles, but also reacted with Ti, forming a titanium aluminide intermediate phase.

Moreira et al. [64] successfully produced high-Cr white cast iron specimens reinforced with TiC particles using powder compacts of Ti (43 µm), Al (12 µm), and graphite (43 µm). The authors reported an effective infiltration of the molten metal and good adhesion between the composite and the base metal. They confirmed the presence of TiC, Cr-rich carbides (M_7_C_3_), and martensite phase in the reinforcement. The average content of spherical TiC particles (with a size lower than 1.34 µm), was 24%. This microstructure led to a hardness increase of 38% and a wear rate decrease of about 30% from that of the high-Cr white cast iron [75].

Two studies were undertaken on the effects of Al content [65] and the size of B_4_C particles [44] in the characteristics of the composite produced. Jiang et al. [65] found that the addition of 30 wt.% Al in the initial mixture of Al-Ti-B_4_C resulted in an increase of seven points of HRC hardness and a decrease in wear volume loss of 23% compared to the base metal (high-Cr alloy steel). Zou et al. [44] produced different composites from Al-Ti-B_4_C powders by varying the particle size of B_4_C and found an inverse relationship between the particle size and the T_c_. The T_c_ affected the type of phases formed: TiC, TiB_2_, and FeAl_x_ were reported for mixtures using small particles (≤28 µm), and an additional phase (Fe_2_B) was reported for mixtures with larger particles (≥40 µm). The authors also verified a decrease in the amount and size of TiC and TiB_2_ particles with the decrease in the T_c_.

Table 7 provides a summary of the main aspects that have been studied using the Al-Ti-C and Al-Ti-B_4_C powder systems for reinforcing steel and iron castings.

## 8. Conclusions

Based on the literature review, the following issues should be highlighted:

- The systems Ti-C, Ni-Ti-C, Ni-Ti-B_4_C, Fe-Ti-C/Fe-Cr-Ti-C, Cu-Ti-B_4_C, Al-Ti-C, and Al-Ti-B_4_C, have been the most used to produce metal matrix composites (MMCs) by casting route, with variable success. Most of the applications were developed in steel parts using the Fe-Ti-C/Fe-Cr-Ti-C powders systems and pressureless infiltration.
- In situ reinforcements are prepared following a common procedure that involves the mixture and compaction of metallic and non-metallic powders in a pre-form, which is inserted in the mold cavity before casting of the molten metal.
- In situ reinforcements are formed from self-propagating combustion reactions activated by the heat of the liquid metal that causes the synthesis of the ceramic phase.
- Variation of the process parameters such as compaction pressure, use of a binder, and initial powders ratio has been performed to improve the hardness and wear performance of the MMC produced.
- The development of TiC-MMCs using in situ methods allows the production of reinforcements up to five times harder than the base metal and three times more resistant to wear, thus achieving a high wear performance material.
- Despite the numerous studies that have been conducted till now, further research is needed to better understand the influence of the microstructural phases of TiC-MMCs on their mechanical properties and wear behavior.

## Figures and Tables

**Table 1:** Adiabatic temperature (T_ad_) of carbides and borides (adapted from [32,34]).

Compound	T_ad_ (°C)
TiC	2940
WC	730
SiC	1530
B_4_C	730
TiB_2_	2920

**Table 2:** Summary of experimental conditions and main results of SHS/casting processing using Ti-C powder system.

Materials and Methods				Results
Base Metal	Reinforcing Materials	Method	Processing Conditions	
Steel [51]	Ti (99.8%; 44 µm) Graphite (99.99%; 44 µm) Alcohol At.r. Ti:graphite—1:1 Wt.r. powders to alcohol— 1:2	Casting with Ar atmosphere	Mixing—24 h Drying—300 °C/10 min Pouring temperature—1550 °C	Reinforcement thickness: 550 µm–1200 µm
				Hardness: 565–610 HV (matrix) 700–1134 HV (reinforcement)
Cast iron [53]	Ti (99.98%, 44 µm) Graphite (99.99%, 30 µm) At.r. Ti:graphite: 1:1 m.wt.—0.01, 0.02, 0.03, 0.04 kg	Casting with Ar atmosphere (5 × 10^−2^ MPa)	Mixing—24 h Drying—412 °C/10 min	Reinforcement thickness: 1 mm (m.wt. 0.01 kg) 6 mm (m.wt. 0.02 kg) 10 mm (m.wt. 0.03 kg) 15 mm (m.wt. 0.04 kg)
Low-carbon steel [50]	Ti (99.98%; 44 µm) Graphite (99.99%; 44 µm) At.r. Ti:graphite 1:1	Infiltration casting	Mixing—24 h CP—500 MPa Pouring temperature—1600 °C	Hardness: 175 ± 4 HV 30 (base alloy) 696 ± 201 HV 30 (reinforcement)
Low-carbon steel [52]	Ti (99.42%, 44 µm)— 80 wt.% Graphite (99.99%, 44 µm)— 20 wt.%	Infiltration casting	Mixing—24 h CP—250, 300, 500, 600 MPa Pouring temperature—1625 °C	Hardness: 326 ± 23 HV 30 (base alloy) 489 ± 116 HV 30 (CP 250 MPa) 1034 ± 88 HV 30 (CP 300 MPa) 1081 ± 234 HV 30 (CP 500 MPa) 1523 ± 290 HV 30 (CP 600 MPa)
Low-carbon steel [56]	Ti (99.98%, 44 µm) Graphite (99.99%, 44 µm) At.r. Ti:graphite—1:1	Infiltration casting	Mixing in a horizontal axle mixer—24 h CP—250, 600 MPa Pouring temperature—1600 °C	Wear mass loss: 0.32 g/16 h (CP 250 MPa) 0.13 g/16 h (CP 600 MPa)
				Hardness: 489 ± 116 HV (CP 250 MPa) 1523 ± 290 HV (CP 600 MPa)
Steel [57]	Ti (99.95%, 44 µm) Graphite (96%, 3 µm) At.% Ti:graphite—55:45 2, 5, 10% aqueous solutions of CMC Wt.r. powders to binder— 2:1	Infiltration casting	Pouring temperature 1550 °C	Wear volume index: 315 mm^3^N^−1^m^−1^ (steel) 50 mm^3^N^−1^m^−1^ (2% CMC) 62 mm^3^N^−1^m^−1^ (5% CMC)
				Hardness: 400 HV 1 (steel) 800 HV 1 (2% CMC) 625 HV 1 (5% CMC)

At.r.—atomic ratio; CP—compaction pressure; Wt.r.—weight ratio; m.wt.—mixture weights.

**Table 3:** Summary of experimental conditions and main results of SHS/casting processing using Ni-Ti-C and Ni-Ti-B_4_C powder systems.

Materials and Methods				Results
Base Metal	Reinforcing Materials	Method	Processing Conditions	
Medium- carbon steel [61]	Ni (99.8%; ≈5 µm) 10, 20, 30, 40 wt.% Ti (99.5%; ≈15 µm) B_4_C (98.0%; 3.5 µm) Stoichiometric— 2TiB_2_-TiC	Infiltration casting	Mixing with Ar atmosphere in a stainless-steel jar—50 rpm/8 h CP—80–85 MPa (70 ± 5% theoretical density) Drying—300 °C/3 h Pouring temperature—1600 °C	Hardness: <20 HRC (matrix) 44 HRC (10 wt.% Ni) 43 HRC (20 wt.% Ni) 48 HRC (30 wt.% Ni) 47 HRC (40 wt.% Ni)
High-carbon steel [59]	Ni (99.8%; ≈5 µm) 10–30 wt.% Ti (99.5%; ≈15 µm) C (99.9%; ≈38 µm) At.r. Ti:C—1:1	Infiltration casting	Mixing in a ball milling—6 h CP—80–85 MPa (70–80% theoretical density) Pouring temperature—1600 °C	Wear volume loss: 2.311 × 10^−10^ m^3^m^−1^ (steel) 0.514 × 10^−10^ m^3^m^−1^ (10 wt.% Ni)
				Hardness: 34 HRC (steel) 57 HRC (10 wt.% Ni)
Austenite Mn-steel [60]	Ni (99.5%; 45 µm)— 40 wt.% (6 g) Ti (99.5%; ≈25 µm) 6.48, 6.65, 6.82 g B_4_C (98.0%; ≈25 µm) 2.52, 1.93, 1.33 g Graphite (99.5%; ≈38 µm) 0, 0.42, 0.85 g	Infiltration casting	Mixing in a ball milling—8 h Drying—300 °C/3 h Pouring temperature—1500 °C	Wear volume loss: 2.281 × 10^−10^ m^3^m^−1^ (steel) 0.5463 × 10^−10^ m^3^m^−1^ (specimen 1—6.48 g Ti; 2.52 g B_4_C) 0.7713 × 10^−10^ m^3^m^−1^ (specimen 2—6.65 g Ti; 1.93 g B_4_C; 0.42 C) 1.1406 × 10^−10^ m^3^m^−1^ (specimen 3—6.82 g Ti; 1.33 g B_4_C; 0.85 C)
				Hardness: 20 HRC (steel) 46 HRC (specimen 1) 43 HRC (specimen 2) 40 HRC (specimen 3)
Mn-steel [58]	Ni (99.5%; 45 µm)— 20 wt.% Ti (99.5%; 25 µm)— 64 wt.% Graphite (99.5%; ≈150, ≈38 and ≈1 µm)— 14 wt.%	Infiltration casting	Mixing—8 h Green densities of 75 ± 2% (theoretical density)	Wear volume loss: 2.268 × 10^−10^ m^3^m^−1^ (steel) 1.8771 × 10^−10^ m^3^m^−1^ (C: ≈150 µm) 1.4014 × 10^−10^ m^3^m^−1^ (C: ≈38 µm) 0.7152 × 10^−10^ m^3^m^−1^ (C: ≈1 µm)
				Hardness: <20 HRC (steel) 40 HRC (C: ≈38 µm) 45 HRC (C: ≈1 µm)

At.r.—atomic ratio; CP—compaction pressure.

**Table 4:** Summary of experimental conditions and main results of SHS/casting processing using Fe-Ti-C powder system.

Materials and Methods				Results
Base Metal	Reinforcing Materials	Method	Processing Conditions	
Ferritic- pearlitic ductile iron [54]	Ti (99.98%; 44 µm) Graphite (99.99%; 44 µm) At.r. Ti:graphite—1:1 Fe (99.4%; 44 µm) 0 (M100), 10 (M90), 50 (M50) wt.%	Infiltration casting	Drying—100 °C/1 h CP—500 MPa Pouring temperature— 1450 °C	Hardness: 742 ± 163 HV (M100) 562 ± 29 HV (M90) 256 ± 38 HV (M50)
High Mn-steel [22]	Ti (30–50 µm) C (30–50 µm) 10, 20, 30, 40, 50 wt.% At.r. Ti:C—4:1 Ti + C: 90, 80, 70, 60, 50 wt.% Fe (40–60 µm) 10, 20, 30, 40, 50 wt.% PVA glue (2%)—3 wt.%	Infiltration casting (lost model)	CP—200 MPa	Wear rate: 40% relative wear rate (Ti + C—80 wt.%)
				Hardness: 48 HRC (Ti + C—80 wt.%)
Mn-steel [48]	Ti (30–50 µm) C (30–50 µm) At.r. Ti:C—4:1 Ti + C: 50, 60, 70, 80, 90 wt.% Fe (40–60 µm) 10, 20, 30, 40, 50 wt.% TiC (1–10 µm) PVA glue (2%)—3 wt.% Sodium silicate Water	Infiltration casting (lost model)	Mixing in a planetary tank—1 h Density of the compacts 1.80, 2.51, 2.79, 3.05, 3.39, 3.58 g∙cm^−3^ Pouring temperature— 1560 °C	Relative wear rate: 22% (density—3.05 g∙cm^−3^— CP 200 MPa; 80 wt.% Ti + C)
				Hardness: ≈13 HRC (base metal) 63 HRC (density—3.05 g∙cm^−3^—CP 200 MPa; 80 wt.% Ti + C)
Gray cast iron [24]	Fe (99.9%; <75 µm)—45 wt.% Ti (99.5%; <75 µm)—51.76 wt.% C (99.5%; <30 µm)—3.24 wt.% PVAL solution (glue) Wt.r. powders:glue—3:1	Pressure-driven infiltration (lost model)	Mixing in a ball mill with a wt.r. ball:powder—10:1— 6 h Drying—50 °C/24 h Vacuum degree—0.06 MPa Pouring temperature— 1450 °C; 1500 °C; 550 °C; 1600 °C	Hardness: 51 HRC (1450 °C) 68 HRC (1500 °C) 61 HRC (1550 °C) 55 HRC (1600 °C)
Low-carbon steel [20]	Ti (99.8 wt.%, 44 µm) Graphite (99.9 wt.%, 44 µm) At.r. Ti:graphite—1:1 Fe (99.8 wt.%, 44 µm) 0, 10, 30, 50, 70 wt.%	Infiltration casting	Mixing in a shaker mixer— 6 h CP—500 MPa Compacts fixed in the mold cavity using a ceramic glue Pouring temperature— 1497 °C	Hardness: 500 HV 30 (with no Fe powder) 350 HV 30 (10 wt.% Fe) 400 HV 30 (30 wt.% Fe) 380 HV 30 (50 wt.% Fe) 250 HV 30 (70 wt.% Fe)

At.r.—atomic ratio; CP—compaction pressure; Wt.r.—weight ratio.

**Table 5:** Summary of experimental conditions and main results of SHS/casting processing using Fe-Cr-Ti-C powder system.

Materials and Methods				Results
Base Metal	Reinforcing Materials	Method	Processing Conditions	
Medium- carbon steel [71]	Low-melting-point compounds Cr—25–28 wt.% Ni—15–30 wt.% C—4–5 wt.% Fe—balance Carbide-forming compounds Ti—27.9 wt.% Si—1.03 wt.% Al—2.12 wt.% Graphite—6.5 wt.% Fe—balance Ratio low-melting-point compounds to the carbide-forming compounds—1:1	Infiltration casting	Mixing in a ball mill—24 h CP—500 MPa Drying—500 °C/2 h Pouring temperature— 1600 °C	…
Medium Mn-steel [9]	High ferrotitanium (25–53 µm) Low ferrotitanium (25–53 µm) At.r. Ti:C—1:1 Graphite (99.9 wt.%, 53 µm)	Infiltration casting	Mixing in a stainless-steel jar—50 rpm/24 h CP—80 MPa Drying—120 °C/5 h	Weight loss: 0.0304 g (quenched Mn13 steel) 0.0162 g (reinforced zone)
				Hardness: 18 HRC (quenched Mn13 steel) 55 HRC (reinforced zone)
Medium- carbon steel [72]	Ti (50–60 µm) C (150–160 µm) Steel powder (35–40 µm) 0 and 20 wt.%	Pressure- driven infiltration (lost model)	Density of 50% Painted 1 mm thickness Drying—50 °C Vacuum degree— 0.065–0.060 MPa	…
Medium- carbon steel [10]	Ti (99.98%; 45 µm) Graphite (98%; 10 µm) At.r. Ti:graphite—1:1 Ti + graphite: 30 wt.% White cast iron powder (3,6 C; 2,2 Si; 0,8 Mn; 5,5 Ni; 10 Cr; 0,5 Mo; Fe bal.)—70 wt.%	Infiltration casting	Mixing—6 h Pouring temperature—1550 °C	Wear rate: 2.8 × 10^−6^ mm^3^N^−1^m^−1^ (reinforcement)
				Hardness: 500 HV 1 (base alloy) 1500 HV 1 (reinforcement)
Medium- carbon steel [62]	Ti (>99.95%, 45 µm) Graphite (>96%, 5 µm) At.r. Ti:graphite—1:1 Hadfield steel powder (moderator)—70 and 90 wt.%	Infiltration casting	Mixing—6 h CP—550 MPa Pouring temperature—1625 °C	Wear rate: 803.90 × 10^−6^ mm^3^N^−1^m^−1^ (base metal) 15.30 × 10^−6^ mm^3^N^−1^m^−1^ (70 wt.% moderator) 48.81 × 10^−6^ mm^3^N^−1^m^−1^ (90 wt.% moderator)
				Hardness: Increase in hardness ranging from 200 to 300 HV) 785 HV 30 (70 wt.% moderator) 580 HV 30 (90 wt.% moderator)
Medium- carbon steel [7]	Ti (99.95%, 44 µm) Graphite (>96%, 3 µm) At.r. Ti:graphite—1:1 High-chromium cast iron powder (moderator) 30, 50, 70 and 90 wt.%	Infiltration casting	Drying—150 °C CP—500 MPa Pouring temperature—1550 °C	Reinforcement thickness: 26 mm (30 wt.% moderator) 24 mm (50 wt.% moderator) 21 mm (70 wt.% moderator) 20 mm (90 wt.% moderator)
				Wear rate: 321.13 mm^3^N^−1^m^−1^ (steel) 22.99 mm^3^N^−1^m^−1^ (30 wt.% moderator) 4.26 mm^3^N^−1^m^−1^ (50 wt.% moderator) 4.39 mm^3^N^−1^m^−1^ (70 wt.% moderator) 2.7 mm^3^N^−1^m^−1^ (90 wt.% moderator)
				Hardness: ≈460 HV 1 (steel) ≈725 HV 1 (30 wt.% moderator) ≈775 HV 1 (50 wt.% moderator) 927 HV 1 (70 wt.% moderator) ≈825 HV 1 (90 wt.% moderator)

At.r.—atomic ratio; CP—compaction pressure.

**Table 6:** Summary of experimental conditions and main results of SHS/casting processing using Cu-Ti-B_4_C and Cu-Ti-C powder systems.

Materials and Methods				Results
Base Metal	Reinforcing Materials	Method	Processing Conditions	
Medium- carbon steel [26]	Cu (99.0%; ≈45 µm) 10–60 wt.% Ti (99.5%; ≈38 µm) B_4_C (99.9%; ≈3.5 µm) Stoichiometric 2TiB_2_-TiC	Infiltration casting	Mixing by ball-milling— 35 rpm/8 h Drying—300 °C/3 h Pouring temperature—1500 °C	Wear volume loss: 3.42 × 10^−10^ m^3^∙m^−1^ (steel) 1.17 × 10^−10^ m^3^∙m^−1^ (10 wt.% Cu) 1.09 × 10^−10^ m^3^∙m^−1^ (20 wt.% Cu) 0.92 × 10^−10^ m^3^∙m^−1^ (30 wt.% Cu) 1.35 × 10^−10^ m^3^∙m^−1^ (40 wt.% Cu) 1.97 × 10^−10^ m^3^∙m^−1^ (50 wt.% Cu) 2.35 × 10^−10^ m^3^∙m^−1^ (60 wt.% Cu)
				Hardness: <20 HRC (steel) 50 HRC (10 wt.% Cu) 48 HRC (20 wt.% Cu) 49 HRC (30 wt.% Cu) 46 HRC (40 wt.% Cu) 41 HRC (50 wt.% Cu) 38 HRC (60 wt.% Cu)
Medium Mn-steel [73]	Cu (99.5%, ≈6 µm)—20 wt.% Ti (99.5%, ≈15 µm) C (99.9%, ≈1, 38, 75, 150 µm) At.r. Ti:C—1:1	Infiltration casting	Mixing—6 h Green densities of 70 ± 2% (theoretical density) Drying—150 °C/3 h Pouring temperature—1500 °C	Wear volume loss: 2.28 × 10^−10^ m^3^∙m^−1^ (steel) 2.07 × 10^−10^ m^3^∙m^−1^ (≈150 µm C) 1.52 × 10^−10^ m^3^∙m^−1^ (≈38 µm C) 0.75 × 10^−10^ m^3^∙m^−1^ (≈1 µm C)
				Hardness: <20 HRC (steel) 34 HRC (≈150 µm C) 42 HRC (≈38 µm C) 46 HRC (≈1 µm C)
Mn-steel [6]	Cu (99.5%; ≈3 µm) 10, 30, 50 wt.% Ti (99.5%; ≈38 µm) B_4_C (99.9%; ≈3.5 µm) Mole ratio Ti:B_4_C—3:1	Infiltration casting	Mixing in a stainless-steel container—≈35 rpm/8 h CP—60 MPa Drying—300 °C/3 h Pouring temperature—1500 °C	Hardness: 18 HRC (steel) 50 HRC (10 wt.% Cu) 58 HRC (30 wt.% Cu) 41 HRC (50 wt.% Cu)
Mn-steel [63]	Cu (99.5%, ≈3 µm)—40 wt.% Ti (99.5%, ≈38 µm) B_4_C (99.9%; ≈3.5, ≈45, 150 µm) Mole ratio Ti:B_4_C—3:1	Infiltration casting	Mixing—35 rpm/8 h (65% theoretical density) Drying—300 °C/3 h Pouring temperature—1600 °C	Wear mass loss (80 N): ≈17.8 mg (Mn-steel) ≈4.3 mg (≈3.5 µm B_4_C) ≈5.2 mg (≈45 µm B_4_C) ≈8 mg (≈150 µm B_4_C)
				Hardness: <20 HRC (Mn-steel) 46 HRC (≈3.5 µm B_4_C) 42 HRC (≈45 µm B_4_C) 34 HRC (≈150 µm B_4_C)
Mn-steel [74]	Cu (99.5%, ≈6 µm) 10–50wt.% Ti (99.5%, ≈25 µm) C (99.9, ≈38 µm) At.r. Ti:C—1:1	Infiltration casting	Mixing in a stainless-steel container—6 h Green densities of 65 ± 2% (theoretical density) Drying—300 °C/3 h Pouring temperature—1500 °C	Wear mass loss (110 N): ≈8.5 mg (steel) ≈5.5 mg (10 wt.% Cu) ≈4.8 mg (20 wt.% Cu) ≈4.9 mg (30 wt.% Cu) ≈5.25 mg (40 wt.% Cu) ≈6.75 mg (50 wt.% Cu)
				Hardness: <20 HRC (steel) 47 HRC (10 wt.% Cu) 36 HRC (20 wt.% Cu) 31 HRC (30 wt.% Cu) 29 HRC (40 wt.% Cu) 27 HRC (50 wt.% Cu)

At.r.—atomic ratio; CP—compaction pressure.

**Table 7:** Summary of experimental conditions and main results for SHS/casting processing using Al-Ti-C and Al-Ti-B_4_C powder systems.

Materials and Methods				Results
Base Metal	Reinforcing Materials	Method	Processing Conditions	
High-Cr alloy steel [65]	Ti (99.5%; <25 µm) B_4_C (98.0%; <3.5 µm) At.r. B:Ti—2:1 At.r. C:Ti—1:1 Al (98.4%; <27 µm) 10, 20, 30, 40 wt.%	Infiltration casting	CP—70–75 MPa (65 ± 2% of theoretical values) Pouring temperature—1600 °C	Wear volume loss: 2.071 × 10^−10^ m^3^m^−1^ (steel) 1.595 × 10^−10^ m^3^m^−1^ (30 wt.% Al)
				Hardness: 50 HRC (steel) 57 HRC (30 wt.% Al)
Medium- carbon steel [44]	Al (99%; ≈29 µm)—30 wt.% Ti (99.5%; ≈38–48 µm) B_4_C (97%; 2.5–3.5; 20–28; 28–40; 63–80, 100–125 µm) Mole ratio Ti:B_4_C—3:1	Infiltration casting	Mixing in a stainless-steel container—≈35 rpm/8 h CP—≈60 MPa (green densities of 65 ± 2%)	…
High-Cr white cast iron [64]	Al (99.0%, 12 µm)—20 wt.% Ti (99.5%, 43 µm)—64 wt.% Graphite (99.0%, 43 µm)— 16 wt.%	Infiltration casting	Mixing in a shaker mixer—7 h CP—70 MPa Pouring temperature—1460 °C	Reinforcement thickness: 6 mm
High-Cr white cast iron [75]	Al (99.0%, 12 µm)—20 wt.% Ti (99.5%, 43 µm)—64 wt.% Graphite (99.0%, 43 µm)— 16 wt.%	Infiltration casting	Mixing in a shaker mixer—7 h CP—70 MPa Pouring temperature—1460 °C	Reinforcement thickness: 6 mm
				Wear rate: 1.29 × 10^−6^ mm^3^∙N^−1^∙mm^−1^ (base metal) 9.01 × 10^−7^ mm^3^∙N^−1^∙mm^−1^ (reinforcement)
				Hardness: 579 ± 47 HV 30 (base metal) 797 ± 112 H V30 (reinforcement)

At.r.—atomic ratio; CP—compaction pressure.
